# Mechanism Underlying a Brief Cognitive Behavioral Treatment for Head and Neck Cancer Survivors with Body Image Distress

**DOI:** 10.21203/rs.3.rs-3303379/v1

**Published:** 2023-09-06

**Authors:** Evan M. Graboyes, Emily Kistner-Griffin, Elizabeth G. Hill, Stacey Maurer, Wendy Balliet, Amy M. Williams, Lynne Padgett, Flora Yan, Angie Rush, Brad Johnson, Taylor McLeod, Jennifer Dahne, Kenneth J. Ruggiero, Katherine R. Sterba

**Affiliations:** Medical University of South Carolina; Medical University of South Carolina; Medical University of South Carolina; Medical University of South Carolina; Medical University of South Carolina; Corewell Health; VA Office of Research and Development; Temple University; Head and Neck Cancer Alliance; Head and Neck Cancer Alliance; Medical University of South Carolina; Medical University of South Carolina; Medical University of South Carolina; Medical University of South Carolina

**Keywords:** body image, cancer, cognitive behavioral therapy, head and neck cancer, psychosocial oncology, survivorship, telemedicine

## Abstract

**Purpose:**

Body image distress (BID) among head and neck cancer (HNC) survivors is a debilitating toxicity associated with depression, anxiety, stigma, and poor quality of life. BRIGHT (Building a Renewed ImaGe after Head & neck cancer Treatment) is a brief cognitive behavioral therapy (CBT) that reduces BID for these patients. This study examines the mechanism underlying BRIGHT.

**Methods:**

In this randomized clinical trial, HNC survivors with clinically significant BID were randomized to receive 5 weekly psychologist-led video tele-CBT sessions (BRIGHT) or dose-and delivery matched survivorship education (attention control [AC]). Body image coping strategies, the hypothesized mediators, were assessed using the Body Image Coping Skills Inventory (BICSI). HNC-related BID was measured with the IMAGE-HN. Causal mediation analyses were used to estimate the mediated effects of changes in BICSI scores on changes in IMAGE-HN scores.

**Results:**

Among 44 HNC survivors with BID, mediation analyses showed that BRIGHT decreased avoidant body image coping (mean change in BICSI-Avoidance scale score) from baseline to 1-month post-intervention relative to AC (p = 0.039). Decreases in BICSI-Avoidance scores from baseline to 1-month decreased IMAGE-HN scores from baseline to 3-months (p = 0.009). The effect of BRIGHT on IMAGE-HN scores at 3-months was partially mediated by a decrease in BICSI-Avoidance scores (p = 0.039).

**Conclusions:**

This randomized trial provides preliminary evidence that BRIGHT reduces BID among HNC survivors by decreasing avoidant body image coping. Further research is necessary to confirm these results and enhance the development of interventions targeting relevant pathways to reduce BID among HNC survivors.

**Trial Registration:**

This trial was registered on ClinicalTrials.gov identifier NCT03831100 on February 5, 2019.

## Introduction

Head and neck cancer (HNC) and its treatments result in substantial life-altering changes related to facial disfigurement, difficulty swallowing, impaired smiling, and challenges speaking. Because these changes are highly visible and affect daily functions, 75% of HNC survivors express body image concerns^1^ and up to 28% have clinically significant body image-related distress (BID).^2^ BID is a source of devastating psychosocial morbidity including a 6-fold increase in moderate-severe depressive symptoms, an 8-fold increase in moderate-severe anxiety symptoms, and increased rates of social isolation and feelings of stigmatization.^2^ As a result, BID among HNC survivors is associated with reduced quality of life (QOL) and may contribute to the two-fold higher rate of suicide mortality relative to other cancer survivors.^2,3^

Despite the disfiguring and functionally debilitating nature of HNC and its treatment, the development of BID among HNC survivors is variable and not related to the extensiveness of disfigurement.^4,5^ For HNC survivors, the experience of disfigurement and function-related impairment occurs in a highly social context (e.g., others noticing and reacting negatively through staring or hurtful words^6,7^). These distressing body image-related experiences occur as HNC survivors conduct routine life activities that are critical to QOL (e.g., social and occupational engagement). As a result, it is imperative that HNC survivors develop skills to cope with these experiences Patients respond to these challenges in adaptive and maladaptive ways. It is imperative that HNC survivors develop the capacities to distinguish between adaptive and maladaptive coping responses and skills, broaden and strengthen their adaptive skills, and learn to suppress or transform maladaptive coping responses. However, it is thought that maladaptive body image coping may be one of the key mechanisms that predispose HNC survivors to BID.^7–9^

Cognitive behavioral therapy (CBT)-based approaches have emerged as potential treatment paradigms for HNC survivors with BID.^10,11^ BRIGHT (Building a Renewed ImaGe after Head & neck cancer Treatment) is a brief tailored CBT that has demonstrated feasibility, acceptability, telehealth compatibility, and preliminary efficacy in both single-arm^12^ and pilot randomized clinical trial (RCT)^13,14^ settings. BRIGHT was developed to target maladaptive body image coping among HNC survivors. Specifically, BRIGHT provides psychoeducation about the cognitive model of body image; teaches self-monitoring about thoughts, feelings, and body image behaviors; offers cognitive restructuring to identify and challenge unhelpful automatic HNC-related body image thoughts; teaches positive body image coping strategies; and reinforces relapse prevention when managing lifelong BID.

There is consensus that evidence-based psychosocial interventions should be based on theory-based mechanisms of change to avoid pseudoscientific interventions, optimize the effectiveness and efficiency of treatment, and advance the science of psychosocial interventions broadly by identifying psychological factors involved in pathology and health.^15,16^ However, little is known about the mechanism of change underlying CBT for BID, particularly among cancer survivors and/or persons with visible disfigurement.^9,17,18^ To address this gap in understanding, this study aims to examine the mechanism of change underlying BRIGHT on BID among HNC survivors. We hypothesize that a brief, tailored CBT (i.e., BRIGHT) will improve BID among HNC survivors by reducing maladaptive body image coping.

## Methods

### Study Approvals

The study was approved by the institutional review board at the Medical University of South Carolina (MUSC). The CONSORT diagram (**eFigure 1** in **Supplement 1**), methods, and primary and secondary analyses for the RCT have been described in detail.^13,14^ Results are reported according to the Consolidated Standards of Reporting Trials Extension (CONSORT Extension) reporting guidelines for randomized pilot and psychological intervention trials.^19,20^

### Patients and Study Procedures

Study patients were recruited in the outpatient setting from the MUSC HNC clinic during a routine survivorship encounter. Eligible patients were adult HNC survivors with clinically significant BID, as determined by a Body Image Scale score ≥ 10,^21^ who had completed treatment 6-weeks to 12 months prior to accrual. The early post-treatment timepoint for trial eligibility was chosen to reflect the preferences of HNC survivors established during our formative work and was utilized in the preceding single arm-trial.^12^ Patients were excluded if they could not read and write English or if they had a severe psychiatric comorbidity (e.g. psychosis). Following written informed consent and completion of baseline assessments, patients were randomized 1:1 to BRIGHT or attention control (AC) using a permuted block randomization design with block sizes of 4 or 6. Study assessments were performed at baseline and then 1-week, 1-month, and 3-months post-intervention.

### Interventions

The BRIGHT and AC interventions have previously been described in detail.^13^ In brief, BRIGHT is a theory-based,^8,9,22–24^ manualized CBT consisting of 5 weekly 60-minute sessions delivered one-on-one by a licensed clinical psychologist via a video telemedicine platform. BRIGHT session topics include (1) psychoeducation about the cognitive model of body image; (2) self-monitoring about thoughts, feelings, and body image behaviors; (3) cognitive restructuring to identify and challenge unhelpful automatic HNC-related body image thoughts; (4) positive body image coping strategies; and (5) maintenance and relapse prevention. AC is a tele-supportive care intervention consisting of educational videos that address non-body image aspects of HNC survivorship. Following best practices for choosing control groups within behavior change RCTs,^25^ we designed AC to match BRIGHT’s dose (5 weekly sessions) and delivery method (video-based telemedicine) while not providing the behavior change mechanism in BRIGHT. Attention control is a tele–supportive care intervention consisting of educational videos that address non–body image aspects of HNC survivorship in 5 modules: (1) introduction to survivorship, (2) physical treatment toxic effects, (3) psychosocial effects of HNC, (4) health maintenance, and (5) financial toxicity. AC was pretested with HNC survivors and refined to optimize its feasibility, credibility, and relevance. Fidelity and adherence to both arms have previously been described.^13^ AC was not delivered by a mental health professional; adherence to AC was monitored by a member of the research team.

### Study Measures

HNC-related BID was assessed with the IMAGE-HN [Inventory to Measure and Assess imaGe disturbancE–Head and Neck]), a validated patient-reported outcome measure of HNC-related BID.^2,26^ The IMAGE-HN score ranges from 0 to 84, with higher scores representing worse HNC-related BID. Cancer-related BID was measured with the Body Image Scale, a validated measure of BID among oncology patients that has been widely used among patients with HNC.^27,28^ Body Image Scale score ranges from 0 to 30, with higher scores representing worse cancer-related BID.

Body image coping skills, the hypothesized mechanism of change underlying BRIGHT, was measured using the Body Image Coping Skills Inventory (BICSI). The BICSI is a 29-item, validated measure of the cognitive and behavioral responses to manage threats to body image.^22^ The BICSI contains three subscales; (1) Appearance Fixing (altering appearance by covering, camouflaging, or correcting the perceived defect), (2) Avoidance (an attempt to escape or avert stressful body-image situations), and (3) Positive Rational Acceptance (acceptance of the challenging event and positive self-care or rational self-talk about one’s appearance). The score for each subscale is calculated by summing the values for the individual questions and thus ranges as follows: avoidance (0–24), appearance fixing (0–30), and positive rational acceptance (0–33). For each subscale, higher scores indicate greater reliance on that type of body image coping.

### Statistical Analysis

Data collected from the previously described study population were included in all descriptive and causal mediation analyses.^13^ Missing data were minimal. One subject was missing two items on the BICSI-Avoidance Subscale from the baseline assessment; these were imputed as the average of the subject’s other items for that baseline Avoidance assessment. All analyses were conducted using Rstudio (R version 4.2.3). Means and standard deviations were summarized by treatment group for each of the mediator variables of interest at baseline (BICSI-Avoidance score, BICSI-Appearance Fixing score, and BICSI-Positive Rationale Acceptance score) and change in the mediators from baseline to 1-week and baseline to 1-month post-intervention. The timing of the 1-week and 1-month post-intervention mediator assessments were chosen to align analysis of the mediator (cause) temporally prior to the outcome (effects; measured at 1- and 3-months post-intervention). Linear models of change from baseline in BICSI scores at 1 week (1 month) as a function of treatment group were used to provide preliminary evidence the intervention had an effect on its hypothesized target. Causal mediation analyses were likewise conducted using linear regression models to evaluate all direct and indirect pathways. To adjust for the negative correlation between change scores and baseline values,^29^ all linear regression models included baseline mediator values as covariates, and all causal mediation models additionally included baseline outcome variable values. Ignoring baseline values for mediators and outcome measurements could lead to possible confounding and unadjusted models would therefore result in biased analyses.^30^ P-values are reported for two-sided tests of differences in mediator changes from baseline at 1-week and 1-month assessments between BRIGHT and AC from linear models adjusting for all mediators at baseline. Consistent with the pilot trial design, all tests are conducted at an alpha = 0.10 level and 90% confidence intervals (CIs) are reported, as pre-specified in the statistical analysis plan.

Causal mediation analyses were conducted using a simulation approach implemented in the mediation package in R.^31^ Mediator variables were analyzed as changes in the BICSI-Avoidance, BICSI-Appearance Fixing, and BICSI-Positive Rationale Acceptance scores from baseline to 1-week post-intervention and baseline to 1-month post-intervention. Causal models estimating effect of treatment (BRIGHT, AC) on the change in the mediator variable (BICSI subscale score) from baseline were modeled using linear regression, adjusting for all baseline mediators (BICSI subscale scores) and the outcome of interest at baseline (IMAGE-HN and Body Image Scale scores). Similarly causal models estimating effects of treatment group on change in BID from baseline to 1- and 3-month post-intervention were modeled using linear regression, adjusting for baseline BID assessment and all mediators at baseline. A non-parametric bootstrap approach was implemented to construct 90% CIs around estimates of total effect, average direct effect and average causal mediation effect from the mediation models with number of simulations equal to 500.

## Results

Baseline characteristics are shown in **eTable 1 in Supplement 1**. Among the 44 HNC survivors with BID, the median age was 63 years (range, 41–80 years) and 61% (27/44) identified as female. The most common head and neck subsite was the oral cavity (50%; 22/44); 61% of patients (27/44) had stage III/IV HNC, and 61% (27/44) received adjuvant (chemo)radiation. Median time since completion of treatment was 3 months (IQR 2–6 months). The baseline mean IMAGE-HN and Body Image Scale scores were 43 (SD 18) and 17 (SD 6), respectively.

### Effect of BRIGHT on Body Image Coping

Table 1 shows the effect of treatment (BRIGHT, AC) on its hypothesized targets of body image coping (BICSI subscale scores) over time. BRIGHT reduced avoidant body image coping (BICSI-Avoidance scale score) from baseline to 1-week post-intervention (model based change [Δ BICSI] = −2.2; 90% CI −3.8 to −0.5) and baseline to 1-month post-intervention (Δ BICSI = −2.8; 90% CI −4.9 to −0.7) relative to AC as shown by linear regression analyses adjusted for baseline levels of all BISCI subscale scores.

### Mechanism Underlying Effect of BRIGHT on HNC-Related BID

**Figure 1:**
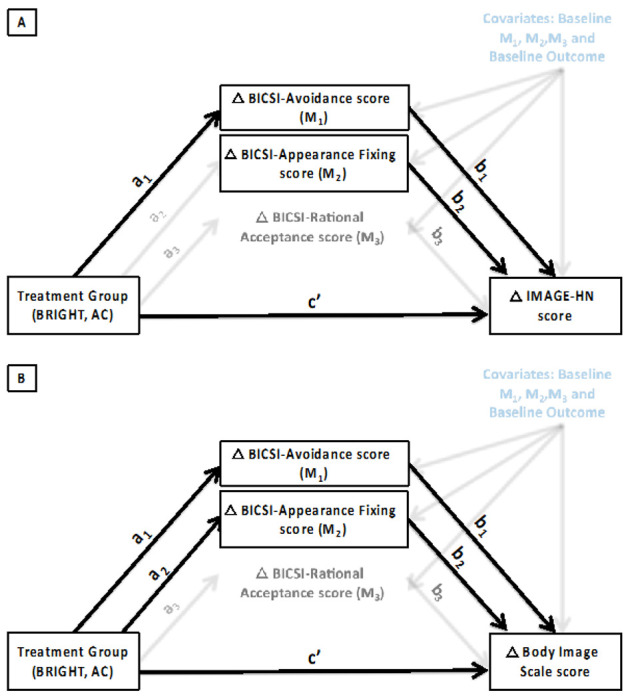
a. Mediation Model for the Effect of BRIGHT on BID Mediation model showing the effect of treatment (BRIGHT, AC) on hypothesized mediators (change in Body Image Coping Skills Inventory scale scores [Avoidance, Appearance Fixing, Rational Acceptance] from baseline to 1-month post-intervention) and outcome (change in mean IMAGE-HN score [**1A**] and change in mean Body Image Scale scores [**1B]**) from baseline to 3-months post-intervention) adjusted for baseline levels of all mediators and the endpoint. Statistically significant paths are indicated with bold arrows. **Abbreviations:** AC = Attention Control; BICSI = Body Image Coping Skills Inventory; BRIGHT = Building a Renewed ImaGe after Head & neck cancer Treatment; IMAGE-HN = Inventory to Measure and Assess imaGe disturbancE–Head and Neck

Table 2 shows the results of the mediation analysis investigating whether the effect of BRIGHT on change in HNC-related BID (IMAGE-HN scores) is mediated through changes in body image coping (BICSI subscale scores). Figure 1A depicts the relationship between treatment (BRIGHT, AC), mediator variables (BICSI-Avoidance [M_1_], Appearance Fixing [M_2_], and Positive Rational Acceptance [M_3_] scores) at 1-month, and the outcome (IMAGE-HN score[I]) at 3 months. The “b_1_” path corresponding to BICSI-Avoidance scale scores (ΔI_M1_ = 1.6; 90% CI 0.6 to 2.7) and the “b_2_” path corresponding to BICSI-Appearance Fixing scale scores (ΔI_M2_ = 1.3; 90% CI 0.6 to 2.0) were both statistically significant, indicating that decreases in avoidant and appearance fixing body image coping from baseline to 1-month post-intervention resulted in decreases in IMAGE-HN scores at 3-months post-intervention. Finally, as shown in Table 2, the effect of BRIGHT on decreases in mean IMAGE-HN scores at 3-months post-intervention was partially mediated by a decrease in BICSI-Avoidance scores at 1-month post-intervention (ΔI = −4.5; 90% CI −9.7 to −0.6).

### Mechanism Underlying Effect of BRIGHT on Cancer-Related BID

Table 3 shows the results of the mediation analysis evaluating the relationship of treatment and body image coping on cancer-related BID (Body Image Scale score [B]). Figure 1B illustrates the relationship between treatment (BRIGHT, AC), mediator variables (BICSI-Avoidance [M_1_], Appearance Fixing [M_2_], and Positive Rational Acceptance [M_3_] scores) at 1-month, and the outcome (Body Image Scale score) at 3 months. The “b_1_” path corresponding to BICSI-Avoidance scale scores (ΔB_M1_ = 0.8; 90% CI 0.4 to 1.2) and the “b_2_” path corresponding to BICSI-Appearance Fixing scale scores (ΔB_M2_ = 0.4; 90% CI 0.1 to 0.7) were both statistically significant, indicating that decreases in avoidant and appearance fixing body image coping from baseline to 1-month post-intervention decreased Body Image Scale scores at 3-months post-intervention. Finally, as shown in Table 3, the effect of BRIGHT on decreases in mean Body Image Scale scores at 3-months post-intervention was partially mediated by a decrease in BICSI-Avoidance (ΔB = −2.3; 90% CI −4.8 to −0.5) and BICSI-Appearance Fixing scale scores (ΔB = −1.3; 90% CI −3.3 to 0.0) at 1-month.

### Explanatory Power of Hypothesized Mechanism

Having shown that the effect of BRIGHT on BID among HNC survivors is partially mediated through improvements in avoidant body image coping, we sought to quantify the explanatory power of the mediation pathway. The change in BICSI-Avoidance scale scores at 1-month explains 26.7% (90% CI: 3.9–67.0%) of the total effect of BRIGHT on change in IMAGE-HN score and 52.8% (90% CI: 13.3–152.0%) of the total effect of BRIGHT on change in Body Image Scale scores at 3 months.

## Discussion

This exploratory study aimed to elucidate the mechanism by which a brief, tailored CBT (BRIGHT) improves BID among HNC survivors. We provide preliminary evidence that BRIGHT effectively reduces maladaptive body image coping relative to AC and demonstrate that the reduction of maladaptive body image coping (particularly avoidance and appearance fixing) decreases BID among HNC survivors. Finally, consistent with our hypothesis, causal mediation analysis in this small sample demonstrated that the mechanism by which BRIGHT improves BID is through decreasing avoidant body image coping.

Multiple meta-analyses have demonstrated that CBT produces durable reductions in BID in patients without visible disfigurement (e.g., eating disorders, body dysmorphic disorder).^32^ However, the theoretical and empirical evidence base supporting CBT for BID in cancer survivors and persons with visible disfigurement is much weaker.^8^ Whereas some studies have suggested that CBT may reduce BID among patients with disfigurement by enhancing body image coping,^18,33,34^ three recent systematic reviews noted methodologic limitations in these studies including non-randomized allocation, comparison to waitlist control, lack of underlying theory, analyses that do not evaluate the mediator in a temporally-relevant manner to ascertain cause and effect, failure to account for mediator-treatment confounding (which breaks the causal inference), and lack of formal mediation analyses.^35–37^

Our study improves upon these methodologic limitations through its randomized design, comparison to AC, theory-based intervention, and causal mediation analysis accounting for multiple time points to demonstrate temporality within the cause and effect pathway and adjustment for baseline variables. These rigorous data extend available evidence and add to the growing literature supporting body image coping strategies as an important mechanism underlying CBT for BID among cancer survivors and persons with visible disfigurement. However, as our analysis showed that the effect of BRIGHT on BID is only partially mediated through a reduction in maladaptive body image coping, other causal mechanisms may be present. Future work should therefore explore other potential mechanisms underlying CBT paradigms (e.g., BRIGHT) effect on body image coping skills such as automatic thoughts, meta-cognitions, or common factors (e.g., therapeutic alliance).^15,38^ In addition, although the BICSI includes one subscale that measures positive, adaptive coping skills (i.e., Positive Rational Acceptance subscale), BRIGHT did not improve this domain relative to AC, and improvements in positive rational acceptance did not mediate improvements in BID. This finding is potentially explained by the lack of theoretical basis for the BICSI Positive Rational Acceptance subscale, which combines items related to acceptance (which may be targeted by acceptance and commitment-based therapies) with items related to rational thinking and automatic thoughts (which may be targeted by cognitive and behavioral-based approaches). Future research in this area should therefore explore the role of improving adaptive body image coping skills in addition to reducing maladaptive body image coping.

Although CBT-based approaches such as BRIGHT have potential as the first evidence-based approach to manage BID among HNC survivors,^12,13^ there are no large RCTs demonstrating the efficacy of any intervention to improve BID in these patients.^28^ Prior studies evaluating interventions to conceal disfigurement^39,40^ and improve self-compassion^41^ showed that these approaches did not decrease BID among HNC survivors. Although there are many potential explanations for the lack of efficacy of these non-CBT-based interventions, one important potential consideration is that they did not target a mechanistically-relevant pathway for the development of BID among HNC survivors. By contrast, our data from both the single-arm and pilot RCT settings provide preliminary support for BRIGHT as the first evidence-based intervention to manage BID among HNC survivors.^12–14^

These mechanism data can now be leveraged to enhance the effectiveness and efficiency of BRIGHT for HNC survivors with BID in the future. By refining BRIGHT to focus more of its therapeutic package (amount of content and/or effectiveness of content delivery) on acquiring adaptive (and reducing maladaptive) body image coping skills, future iterations of BRIGHT could have an even greater effect on reducing BID among HNC survivors. In addition, by removing parts of the BRIGHT therapeutic package that are not mechanistically relevant, we could also enhance the efficiency of BRIGHT, thereby improving scalability, cost-effectiveness, patient and clinician burden, and potentially adoption and sustainability within clinical care.

Although this trial contains a number of strengths including its randomized design, manualized intervention, comparison to dose- and delivery-matched AC, use of validated PROs, and focus on clinically relevant endpoints, findings should be interpreted within the context of several limitations. Consistent with its pilot nature, the study had a small sample size, was conducted at a single-site, was powered for the efficacy analysis (not the mediation analysis), did not control for multiple comparisons, and had a short follow-up duration (3 months post-intervention). We are therefore cautious to overinterpret these encouraging but preliminary findings, which should be considered hypothesis-generating and confirmed in a larger, multi-center trial. Potentially important covariates such as educational attainment and employment status were not collected in the trial and thus could not be analyzed.^2^ Although we measured change in the potential mechanism variable immediately post-treatment, we did not measure changes of the hypothesized mechanism variable during treatment. The BICSI was developed for patients without cancer or visible differences^22^ and thus (1) includes items that are not relevant for cancer survivors (e.g., “I eat something to help me deal with the situation”) and (2) fails to assess image coping skills crucial to patients with cancer (e.g., related to bodily function and functional abilities).^7,24^ Finally, this study did not analyze potentially important mechanisms underlying BRIGHT such automatic thoughts, meta-cognitions, or common factors (e.g., therapeutic alliance).^15,38^

## Conclusions

This pilot RCT provides preliminary evidence supporting the hypothesis that BRIGHT, a tailored brief tele-CBT intervention, improves BID among HNC survivors through a mechanism of decreasing avoidant body image coping. Further research is necessary to confirm these results, explore other potential mechanisms underlying CBT for BID among patients with cancer, and enhance the development of interventions that target mechanistically relevant pathways to reduce BID among HNC survivors.

## Figures and Tables

**Table 1:** Effects of Treatment on Body Image Coping Skills

	No (%)			

	BRIGHT (n = 20)	Attention Control (n = 24)		

Measure^a,b^	Mean Score (SD)	Mean Score (SD)	Model Based Effect Size^c^ 90% CI	P-value^c^
BICSI-Avoidance score	9.1 (4.4)	10.2 (4.0)	−2.2 (−3.8,−0.5)	0.031
Baseline	−2.2 (2.5)	0.5 (3.8)	−2.8 (−4.9,−0.7)	0.029
Δ 1 week	−2.9 (4.3)	0.5 (4.0)		
Δ 1 month				

BICSI-Appearance Fixing score	13.5 (7.1)	15.3 (6.1)	−2.1 (−4.9,0.8)	0.23
Baseline	−1.4 (5.6)	−0.2 (5.5)	−2.6 (−5.4,0.2)	0.13
Δ 1 week	−1.5 (5.4)	0.2 (6.2)		
Δ 1 month				

BICSI-Positive Rational Acceptance score	16.1 (5.0)	16.8 (4.9)	−1.8 (−4.5,0.9)	0.27
Baseline	−0.6 (5.4)	1.0 (5.5)	0.9 (−1.9,3.7)	0.60
Δ 1 week	0.9 (5.0)	0.0 (6.2)		
Δ 1 month				

a Outcomes are measured post-completion of treatment

b For each BICSI subscale score, higher scores indicate greater reliance on that type of skill

c Effect sizes and p-values from models of differences in change in mediator from baseline between treatment groups, adjusting for all three mediators at baseline

Abbreviations: BICSI = Body Image Coping Skills Inventory; BRIGHT = Building a Renewed ImaGe after Head & neck cancer Treatment

**Table 2:** Mediation Analysis Demonstrating the Relationship of Treatment and Hypothesized Mediators on Head and Neck Cancer-Related Body Image Distress (IMAGE-HN Score)

	Effect of BRIGHT on Mediating Variable (a path)		Effect of Mediating Variable on IMAGE-HN score (b path)		Average Direct Effect of BRIGHT on IMAGE-HN score (c’ path)		Total Effect of BRIGHT on IMAGE-HN score (c path)		Average Causal Mediated Effect of BRIGHT on IMAGE-HN score (a x b)	
Mediator^a^	Estimate (90% CI)	p	Estimate (90% CI)	p	Estimate (90% CI)	p	Estimate (90% CI)	p	Estimate (90% CI)	p
Δ BICSI Avoidance score										
Baseline to 1-week	**−2.4** **(−4.0,−0.9)**	**0.013**	**1.6** **(0.4,2.8)**	**0.034**	−5.4 (−11.3,0.5)	0.13	**−9.3** **(−15.3,−3.0)**	**0.019**	**−3.9** **(−8.6,−0.0)**	**0.10**
Baseline to 1-month	**−2.7** **(−4.8,−0.6)**	**0.039**	**1.6** **(0.6,2.7)**	**0.009**	**−12.2** **(−20.4, −3.3)**	**0.024**	**−16.7** **(−24.7,−8.6)**	**0.002**	**−4.5** **(−9.7,−0.6)**	**0.039**
Δ BICSI Appearance Fixing score										
Baseline to 1-week	−2.5 (−5.4,0.3)	0.14	**1.0** **(0.3,1.6)**	**0.023**	**−6.9** **(−12.3,−1.9)**	**0.030**	**−9.3** **(−15.3,−3.0)**	**0.019**	−2.4 (−5.3,0.3)	0.14
Baseline to 1-month	−2.7 (−5.6,0.2)	0.13	**1.3** **(0.6,2.0)**	**0.005**	**−13.2** **(−20.5,−6.2)**	**0.006**	**−16.7** **(−24.7,−8.6)**	**0.002**	−3.5 (−8.1,0.3)	0.15
Δ BICSI Positive Rational Acceptance score										
Baseline to 1-week	−2.0 (−4.8,0.7)	0.22	−0.0 (−0.8,0.7)	0.93	**−9.4** **(−15.5,−3.0)**	**0.019**	**−9.3** **(−15.3,−3.0)**	**0.019**	0.1 (−2.4,2.5)	1.0
Baseline to 1-month	0.5 (−2.3,3.4)	0.76	0.2 (−0.7,0.9)	0.63	**−16.8** **(−24.8,−8.3)**	**0.003**	**−16.7** **(−24.7,−8.6)**	**0.002**	0.1 (−1.6,1.5)	0.99

a Each analysis was conducted evaluating the mediator at 1-week post intervention and the outcome variable (IMAGE-HN) at 1-month post-intervention (top row for each mediator) and mediator at 1-month post intervention and the outcome variable (IMAGE-HN) at 3-months post-intervention (second row for each mediator)

b Each model adjusts for all three mediators at baseline and the IMAGE-HN at baseline

Abbreviations: BICSI = Body Image Coping Skills Inventory; IMAGE-HN = Inventory to Measure and Assess imaGe disturbancE–Head and Neck

**Table 3:** Mediation Analysis Demonstrating the Relationship of Treatment and Hypothesized Mediators on Cancer-Related Body Image Distress (Body Image Scale score)

	Effect of BRIGHT on Mediating Variable (a path)		Effect of Mediating Variable on BIS score (b path)		Average Direct Effect of BRIGHT on BIS score (c’ path)		Total Effect of BRIGHT on BIS score (c path)		Average Causal Mediated Effect of BRIGHT on BIS score (a x b)	
Mediator^a^	Estimate (90% CI)	p	Estimate (90% CI)	p	Estimate (90% CI)	p	Estimate (90% CI)	p	Estimate (90% CI)	p
Δ BICSI Avoidance score										
Baseline to 1-week	**−2.4** **(−4.0,−0.7)**	**0.021**	**0.9** **(0.4,1.3)**	**0.003**	0.4 (−2.6,3.1)	0.88	−1.7 (−5.0,1.1)	0.31	**−2.1** **(−4.1,−0.5)**	**0.024**
Baseline to 1-month	**−2.9** **(−5.1,−0.8)**	**0.026**	**0.8** **(0.4,1.2)**	**0.002**	−2.0 (−5.0,1.1)	0.28	**−4.3** **(−7.6,−1.2)**	**0.016**	**−2.3** **(−4.8,−0.5)**	**0.019**
Δ BICSI Appearance Fixing score										
Baseline to 1-week	**−3.3** **(−5.8,−0.8)**	**0.035**	**0.4** **(0.1,0.7)**	**0.042**	−0.4 (−3.3,2.3)	0.76	−1.7 (−5.0,1.1)	0.31	**−1.3** **(−2.9,0.2)**	**0.054**
Baseline to 1-month	**−3.1** **(−6.0,−0.3)**	**0.069**	**0.4** **(0.1,0.7)**	**0.027**	**−3.0** **(−5.7,−0.2)**	**0.078**	**−4.3** **(−7.6,−1.2)**	**0.016**	**−1.3** **(−3.3,−0.0)**	**0.086**
Δ BICSI Positive Rational Acceptance score										
Baseline to 1-week	−2.1 (−4.9,0.6)	0.20	0.0 (−0.3,0.4)	0.83	−1.6 (−5.0,1.3)	0.36	−1.7 (−5.0,1.1)	0.31	−0.1 (−1.2,0.9)	0.92
Baseline to 1-month	0.7 (−2.2,3.6)	0.70	0.2 (−0.1,0.5)	0.33	**−4.4** **(−7.6,−1.2)**	**0.024**	**−4.3** **(−7.6,−1.2)**	**0.016**	0.1 (−0.8,0.9)	0.90

a Each analysis was conducted evaluating the mediator at 1-week post intervention and the outcome variable (BIS) at 1-month post-intervention (first row for each mediator); and mediator at 1-month post intervention and the outcome variable (BIS) at 3-months post-intervention (second row for each mediator)

b Each model adjusts for all three mediators at baseline and the BIS score at baseline

Abbreviations: BICSI = Body Image Coping Skills Inventory; BIS = Body Image Scale

## Data Availability

We will make de-identified data available to users under a data-sharing agreement that provides for: (1) a commitment to using the data only for research purposes and not to identify any individual participant; (2) a commitment to securing the data using appropriate technology; and (3) a commitment to destroying or returning the data after analyses are completed. All data sharing will comply with privacy and confidentiality protections such as the NIH Certificate of Confidentiality and applicable laws, regulations, and policies governing data derived from human participants. Data requests should be addressed to the corresponding author at graboyes@musc.edu
